# Lack of Evidence for an Association between *Iridovirus* and Colony Collapse Disorder

**DOI:** 10.1371/journal.pone.0021844

**Published:** 2011-06-30

**Authors:** Rafal Tokarz, Cadhla Firth, Craig Street, Diana L. Cox-Foster, W. Ian Lipkin

**Affiliations:** 1 Center for Infection and Immunity, Mailman School of Public Health, Columbia University, New York, New York, United States of America; 2 Department of Entomology, The Pennsylvania State University, University Park, Pennsylvania, United States of America; Martin-Luther-Universität Halle, Germany

## Abstract

Colony collapse disorder (CCD) is characterized by the unexplained losses of large numbers of adult worker bees (*Apis mellifera*) from apparently healthy colonies. Although infections, toxins, and other stressors have been associated with the onset of CCD, the pathogenesis of this disorder remains obscure. Recently, a proteomics study implicated a double-stranded DNA virus, invertebrate iridescent virus (Family *Iridoviridae*) along with a microsporidium (*Nosema* sp.) as the cause of CCD. We tested the validity of this relationship using two independent methods: (i) we surveyed healthy and CCD colonies from the United States and Israel for the presence of members of the *Iridovirus* genus and (ii) we reanalyzed metagenomics data previously generated from RNA pools of CCD colonies for the presence of *Iridovirus*-like sequences. Neither analysis revealed any evidence to suggest the presence of an *Iridovirus* in healthy or CCD colonies.

## Introduction

Honey bee decline has become an increasingly important problem worldwide, and has been attributed to multiple underlying causes [1], [2]. A significant contributor to colony losses is Colony Collapse Disorder (CCD), which is characterized by the sudden absence of adult honey bees (*Apis mellifera*) in hives, while both resources and brood remain. Since the initial description of CCD in 2006, annual colony losses in the United States have exceeded 30%, a number significantly greater than previous average losses [1]. The percentage of collapsed CCD colonies has declined in the last five years; however, approximately one quarter of beekeepers continue to report CCD symptoms in more than 60% of their annual colony losses [3].

Although CCD continues to be associated with an annual colony loss of approximately 10% in the United States [1], attempts to identify the cause or combination of causes that result in CCD have not been successful. Although initial efforts focused on identifying a single pathogen, a consensus has recently emerged that multiple pathogens in concert with increasing environmental stressors are likely to be the driving factors behind CCD [4], [5], [6], [7]. Several pathogens in particular have repeatedly been identified in concert with the appearance of CCD, including the microsporidium *Nosema ceranae* [8] and members of the *Picornavirales* such as Israeli acute paralysis virus [9].

Recently, a double-stranded DNA virus (dsDNA) belonging to the *Iridovirus* genus (Family *Iridoviridae*) has been proposed to act synergistically with *N. ceranae* to cause the development of CCD [8]. In an attempt to use an unbiased proteomics approach for the detection of CCD markers, Bromenshenk et al. [8] identified 139 peptide fragments with homology to invertebrate iridescent virus 6 (IIV-6), the only member of the genus for which full genome sequences and proteomic analysis are available [10]. IIV-6 naturally infects a range of lepidopteran species, but has not been shown to infect any hymenopteran in the wild [11]. However, another invertebrate iridescent virus (IIV-24) has previously been associated with disease in the Eastern honey bee (*A. cerana*) [12]. Unfortunately, only 355 bp of IIV-24 sequence is publicly available, making it difficult to determine if the peptides identified as IIV-6 by Bromenshenk et al. [8] could be more closely related to IIV-24. Notably, in a re-interpretation of this proteomic analysis, Foster [13] suggested instead that the peptides identified as IIV-6 have higher similarity to *A. mellifera* protein sequences. To reconcile this discrepancy and test the intriguing hypothesis proposed by Bromenshenk et al., we surveyed CCD and healthy *A. mellifera* colonies from the United States and Israel for the presence of several invertebrate iridescent viruses, and re-analyzed previously generated metagenomic data from CCD colonies for *Iridovirus* sequences.

## Methods

One hundred and sixty-three bees were surveyed for the presence of members of the *Iridovirus* genus. These were collected in 2007–2009 from 35 colonies distributed across nine commercial apiaries, seven of which have been previously described [5], [9]. All samples were obtained under the auspices of the USDA and the Pennsylvania Department of Agriculture. Four colonies appeared healthy at the time of collection and the remaining 31 were in various stages of collapse, as described in Cox-Foster et al. [9]. Many of these apiaries were also represented by the colonies sampled by Bromenshenk et al. [8]. Samples were stored at −70°C from the time of collection.

Invertebrate iridescent viruses have been shown to replicate primarily in the gut walls, Malpighian tubules and fat-bodies of bees [12]; therefore, total nucleic acid was extracted from individual bee abdomens using the EasyMag Extraction platform (Biomerieux). Four assays were developed to detect the presence of invertebrate iridescent viruses. First, a quantitative SYBR Green PCR assay targeting conserved regions of the DNA-dependent RNA polymerase of IIV-6 (based on GenBank accession number NC_003038) was performed in duplicate (Table 1). Two nested PCR assays targeting conserved regions of the DNA Polymerase gene were also designed. The first assay was specific for IIV-6 (Table 1). In an attempt to capture more of the diversity present in the *Iridovirus* genus, a second nested assay was developed based on an alignment of IIV-6 and IIV-31 (GenBank accession number AJ279821), the only members of the *Iridovirus* genus with available DNA polymerase sequence data. Finally, an assay targeting the only sequence available for IIV-24 (GenBank accession number AF042340) was developed in a highly conserved region of the capsid. PCR assays were performed in 20 µl reactions containing 0.25 µM of each primer, 0.125 mM dNTPs, and 1 mM MgCl_2_. The primer annealing temperatures for each assay are given in Table 1. Plasmids containing the relevant portion of IIV-6 resuspended in 25 ng/µl of bee genomic DNA and DNA extracted from IIV-31-positive isopods (*Armadillidium vulgare)* were used as positive controls. The IIV-24-specific assay was also able to detect the IIV-6 positive control. The sensitivity of each assay was evaluated by serial dilution of the IIV-6 plasmid standard. To verify the quality of our extracted nucleic acid, we also determined the copy number of the bee nuclear genome present using a *β-actin* quantitative assay [14].

**Table 1:** Sequences of the primers used for the detection of members of the genus *Iridovirus.*

Primer name	Primer sequence (5′ to 3′)	Product length (nt)	Annealing Temperature (°C)
IIV6.DdRp.F*	CCCAGCATACTATAACATGTCTGCAA	94	60
IIV6.DdRp.R*	GAATATCCTCTGACGACATCATTCC		
IIV6.DNAPol1.F	GGTGGAATGGGATGATCATG	270	57
IIV6.DNAPol1.R	CCCCCTATATAACCAGGAGTTTG		
IIV6.DNAPol2.F	GATGTGTTCGTATCTTGTGATGG	120	53
IIV6.DNAPol2.R	GGGCCAGGAGTTTGTTTATTAAC		
IIV24.CP.F	GGATATGACAATATGATTGGAAAT	177	50
IIV24.CP.R	CGTTAATTTGCATTTCATTGTA		
IIV.DNAPol1.F	GGGGARTTYTWCCSACAATC	283	50
IIV.DNAPol1.R	GGCTWCCCATRTAWGTKGTRCACA		
IIV.DNAPol2.F	GGGATCTTYTRGAYGCCAGAA	227	54
IIV.DNAPol2.R	GGCATAAAYGGTAAMGCTCCAAC		

*Primers for SYBR Green Assay.

Following the report of an IIV-6-like virus present in samples from the same colonies surveyed by Cox-Foster et al. in 2007 [9], we reanalyzed the metagenomic data generated in the 2007 study for the presence of invertebrate iridescent viruses. The purported identification of IIV-6 in these samples was based upon proteomic analysis, suggesting the presence of mRNA transcripts that should be detectable by a metagenomics approach. A searchable database was first created using all 1204 protein-coding sequences for the *Iridovirus* genus (GenBank Tax ID: 10487) and a homology search with blastx [15] was performed using individual reads previously generated from four pools of RNA [9].

## Results and Discussion

PCR assays were used to test for the presence of members of *Iridovirus* in healthy and CCD colonies. All four assays were optimized to detect 1–10 DNA copies. We found no evidence of *Iridovirus* in any of the 163 bees, despite detecting an average of 10^5^–10^6^ DNA copies of *β*-*actin* per microliter in these samples. During the initial characterization of IIV-24 in diseased bees (*A. cerana*), it was estimated that each bee contained an average of 10^10^–10^11^ viral particles [12], a quantity likely to be well within the range of detection of our PCR assays. The design of our assays was limited by both the paucity of *Iridovirus* sequences publicly available and the absence of any nucleic acid or amino acid sequences linked to the *Iridovirus* identified by mass spectrometry [8]. To address these deficiencies, we targeted highly conserved regions of the genome and used multiple PCR assays designed to detect IIV-6 or an IIV-6 related virus, as well as IIV-24. We focused on IIV-6-related viruses because of the reported presence of 139 IIV-6 peptides in 100% of CCD and 75% of healthy colonies [8]. We also targeted IIV-24, as it is the only *Iridovirus* previously associated with disease in bees [12], [16]. Nevertheless, none of the assays revealed IIV-6 or IIV-24-like viruses in bees from healthy or CCD colonies.

The results of our reanalysis of the metagenomic data from earlier studies of CCD colonies [9] also does not suggest the presence of an *Iridovirus* in CCD bees. Using an e-value cut-off set at 0.0001, two of the four RNA pools (representing pools of CCD positive samples) did not reveal any sequences with homology to any *Iridovirus*. The third and fourth pools, comprised of samples from healthy colonies and royal jelly, contained seven reads with low similarity to IIV-6 (e-value > 1e^−18^). Blasting these individual reads against the entire non-redundant protein-coding database revealed a higher similarity to bacterial or bee genomic sequence in all cases (e-values from 2–88 orders of magnitude lower than when the search was restricted to *Iridovirus*). Although these results do not exclude the possibility that *Iridovirus* sequences may have been present at extremely low concentrations in the 2007 sample set, they do not lend support to a linkage between invertebrate iridescent viruses and CCD.

Typical signs of infection with invertebrate iridescent viruses include lethargy, early and rapid mortality and in bees, the loss of flight [17], [18]. The absence of adult worker bees in otherwise healthy colonies that characterizes CCD does not appear to be consistent with signs of invertebrate iridescent virus infection. Furthermore, a primary characteristic of pathogenic invertebrate iridescent virus infections is the presence of a purple-blue iridescence that can be observed both in centrifuged viral particles and directly in infected tissues by microscopy (fat body, Malpighian tubules and the gut wall), even in mild infections [12], [17], [18], [19]. Presumably, any iridovirus present at a level high enough to initiate colony collapse (even if the presence of *N. ceranae* is also required) would also be present at levels high enough for the detection of iridescence. However, during the examination of bee tissues from a large number of CCD colonies, no iridescent qualities were observed (data not shown).

Admittedly, the possibility exists that the invertebrate iridescent virus detected by mass spectrometry is unrelated to those targeted here, although we consider this to be unlikely. Indeed, it has been suggested that the peptides identified as IIV-6-like by Bromenshenk et al. [8] actually show higher similarity to *A. mellifera* proteins when the host proteome is included in the analysis [13]. When our data is considered along with that of Foster [13] it therefore seems unlikely that an invertebrate iridescent virus related to IIV-6 or IIV-24 is a significant contributor to CCD.
